# A novel approach for reliable qualitative and quantitative prey spectra identification of carnivorous plants combining DNA metabarcoding and macro photography

**DOI:** 10.1038/s41598-022-08580-8

**Published:** 2022-03-21

**Authors:** Thilo Krueger, Adam T. Cross, Jeremy Hübner, Jérôme Morinière, Axel Hausmann, Andreas Fleischmann

**Affiliations:** 1 School of Molecular and Life Sciences, Curtin University, Bentley, Australia; 2 EcoHealth Network, Brookline, MA USA; 3 Zoologische Staatssammlung München (SNSB-ZSM), Munich, Germany; 4 Advanced Identification Methods - AIM GmbH, Leipzig, Germany; 5 Botanische Staatssammlung München (SNSB-BSM), Munich, Germany; 6 GeoBio-Center LMU, Ludwig-Maximilians-University, Munich, Germany

**Keywords:** Plant ecology, Community ecology, Molecular ecology

## Abstract

Prey spectra (the number and composition of captured arthropods) represent a crucial aspect of carnivorous plant ecology, yet remain poorly studied. Traditional morphology-based approaches for prey identification are time-intensive, require specialists with considerable knowledge of arthropod taxonomy, and are hampered by high numbers of unidentifiable (i.e., heavily digested) prey items. We examined prey spectra of three species of closely-related annual *Drosera* (Droseraceae, sundews) from tropical northern Australia using a novel DNA metabarcoding approach with *in-situ* macro photography as a plausibility control and to facilitate prey quantity estimations. This new method facilitated accurate analyses of carnivorous plant prey spectra (even of heavily digested prey lacking characteristic morphological features) at a taxonomic resolution and level of completeness far exceeding morphology-based methods and approaching the 100% mark at arthropod order level. Although the three studied species exhibited significant differences in detected prey spectra, little prey specialisation was observed and habitat or plant population density variations were likely the main drivers of prey spectra dissimilarity.

## Introduction

Carnivorous plants are characterised by adaptations to trap, kill and derive nutritional benefit from animal prey^1^. Since they typically grow in soils where nitrogen and phosphorus are limiting, obtaining these macronutrients by means of arthropod prey capture forms an essential component of their survival strategy^2^. This ecological distinction has made them a popular subject of research since the early work of Darwin^3^, yet studies focussing on the number and composition of captured prey (i.e., their prey spectra) remain surprisingly scarce. Only five such studies have been done in Australia, the global centre of carnivorous plant diversity with approx. 250 species^4–8^. Characterising the prey spectra of carnivorous plants is crucial for understanding their requirements for survival.

Where prey spectra of carnivorous plants have been examined, they have traditionally been analysed by collecting samples of their trapping leaves (dry or in alcohol) before identifying captured prey items by morphological features, usually under a stereo microscope (e.g.^4–7,9–14^). This method is extremely time-intensive and requires considerable taxonomic identification skills covering a wide range of arthropod taxa, or help of insect specialists to identify prey items. Identification may also become impossible for heavily digested prey items that have lost crucial diagnostic morphological features^8^. Collecting trapping leaves can also be logistically challenging in remote regions that may only be accessible by air travel during the main growing season of most carnivorous plant taxa, as such travel precludes the usage of alcohol for conserving samples. An alternative approach using *in-situ* macro photography of prey items was tested by Krueger et al.^8^. While this method allowed rapid and non-invasive documentation of carnivorous plant prey spectra data even under extreme field conditions and provided highly accurate data on prey quantity (prey count), a significant proportion (up to 80%^8^) of prey items remained unidentifiable. Additionally, identification below the taxonomic level of arthropod order using only *in-situ* macro photographs proved to be extremely difficult, and was often impossible^8^.

With recent advances in technologies such as high throughput DNA sequencing, DNA metabarcoding has become a promising tool for analysing environmental samples containing diverse and complex arthropod assemblages^15–22^. Here, DNA of all specimens contained in a sample is extracted holistically and amplified using universal barcode primers targeting the mitochondrial cytochrome c oxidase subunit I (CO1-5P) gene^16,23^. After sequencing, each DNA barcode sequence is subsequently compared with reference libraries on curated databases such as BOLD^24,25^ and NCBI GenBank (https://www.ncbi.nlm.nih.gov) to obtain taxonomic identifications. Metabarcoding thus promises to allow for taxonomic resolution at much finer scale and much higher completeness for environmental bulk samples, such as carnivorous plant prey spectra, provided reference sequences from reliably determined voucher specimens of the targeted species are available on these databases. However, metabarcoding usually does not allow for accurate prey quantity estimations (neither total count of individuals nor biomass), as the CO1 marker is not capable of distinguishing between individuals and obtained read count data are affected by the amount of DNA extracted from prey items which is likely highly variable among different prey taxa, sizes and digestion stages^26,27^. Indeed, the only available study analysing prey spectra of carnivorous plants using metabarcoding did not attempt to obtain prey quantity estimates for these reasons^27^. Thus, metabarcoding of carnivorous plant prey assemblages allows for analysis of what is captured (i.e., taxonomic analysis, such as prey composition), but not how much is captured (i.e., prey quantity or biomass or even relative prey abundances). Crucially, metabarcoding approaches also require plausibility controls as they are extremely sensitive and therefore prone to false positive identifications by even minuscule DNA contamination^15,28^.

To study the prey spectra of three closely related Western Australian carnivorous plant species, we developed and evaluated a novel approach combining DNA metabarcoding and *in-situ* macro photography. All three species (*Drosera finlaysoniana*, *D. hartmeyerorum* and *D. margaritacea*) belong to *Drosera* sect. *Arachnopus* and have similar, linear adhesive trapping leaves. The prey spectra of *D. finlaysoniana* and *D. margaritacea* have not been characterised previously, that of *D. hartmeyerorum* was studied by Krueger et al.^8^. This group of sundews is of particular interest for prey spectra research as they are annuals which appear to depend heavily on supplementary nutrition derived from prey capture^29^ and furthermore have evolved highly specific morphological features which have been hypothesised to function as prey attractants, such as trap scent and specialised leaf trichomes^8,30–32^. By using *in-situ* macro photography (as established by Krueger et al.^8^) as a plausibility control to detect false positive identifications or contaminations in the metabarcoding data and to enable the calculation of total prey quantity, we aimed to obtain, for the first time, carnivorous plant prey spectra data of unprecedented taxonomic resolution and completeness. At coarse taxonomic levels, we expected that this new approach would yield prey spectra similar to that previously observed in *D.* sect. *Arachnopus*^8^. We further expected to confirm significant prey spectra differences between study sites^8,27^ and among species with different trapping leaf sizes^8,33^.

## Materials and methods

### Study sites

Plants were sampled at three study sites in Western Australia in July 2020 (Table 1). Sites 1 and 3 featured large plant populations in freshwater lake margin habitats (especially at Site 1 which consisted of an extremely large and high-density population of *D. finlaysoniana*, comprising millions of individuals), while only ca. 100 plants of *D. margaritacea* were found in a small and dry artificial drainage channel at Site 2 (Supplementary Fig. S1). All studied plants were identified by T.K. and voucher specimens (*Krueger 6*, *Krueger 7* and *Krueger 8*) were deposited in the Western Australian Herbarium (PERTH). Collection of plant material complied with relevant institutional, national, and international guidelines and legislation. A flora taking licence (FT61000038-2) was obtained from the Western Australian Department of Biodiversity, Conservation and Attractions. Plant material was exported to Germany for scientific study under export permit WT2020-001235 issued by the Australian Department of Agriculture, Water and the Environment.

**Table 1:** Summary of the three study sites in Western Australia.

Site	Location	Sampling date	Species studied	Number of plant individuals sampled	Number of sampled leaves per individual plant	Number of *in-situ* prey pictures
Site 1	Great Northern Highway, North of Cue	13 July 2020	*D. finlaysoniana*	10	5	98
Site 2	Great Northern Highway, between Derby and Fitzroy Crossing	18 July 2020	*D. margaritacea*	10	5	154
Site 3	Great Northern Highway, between Broome and Derby	19 July 2020	*D. hartmeyerorum*	10	5	195

### Leaf sampling

Ten plants from each population were randomly selected for study, and five randomly chosen leaves per plant were removed with forceps (scientific collection license FT61000038-2). Each sample thus constituted of five leaves belonging to a single individual and there were ten individuals (replicates) per species. Only fully developed, mucilage-secreting (i.e., “active”) leaves were collected as the heavily digested prey items found on old leaves would complicate both quantitative analysis (e.g., by counting fragmented prey items multiple times) and metabarcoding (due to heavily degraded prey DNA). Three species of *D.* sect. *Arachnopus* were studied (N = 30), consisting of ten replicates per species and a total of 150 collected leaves (Table 1).

### In-situ macro photography

Detached sampled leaves were carefully placed on paper sheets (with the tentacle-bearing, sticky, prey-containing side facing upwards so that no prey items would be lost by adhering to the sheets) with a scale (ruler) to record leaf length. Paper sheets were not re-used to prevent cross-contamination among samples. 447 macro photographs of the collected leaves were taken using a digital camera with a macro lens, and total prey was counted for each sample based on these images. All visible prey items were counted, regardless of digestion state. Other plant material sticking to the leaves (such as seed) did not trigger any tentacle motion and was thus easy to distinguish from prey items. While some prey items may have originated from the same captured prey animal (which was subsequently disintegrated), such cases were often uncertain or difficult to reconstruct and therefore always counted as if they were separate prey. The prey count value per sample was defined as the total number of observed prey items on five randomly selected leaves of a single *Drosera* individual. Finally, the strong effect of leaf size on prey counts^8^ was mitigated by calculating prey count values per cm of leaf length (because even within a single individual of *D*. sect. *Arachnopus* leaf size can be highly variable^8^). All three studied *Drosera* species have a narrowly linear-lanceolate leaf shape and prey counts per cm of leaf length thus closely approximate prey counts per leaf area. For leaf length, the arithmetic mean of the five collected leaves was used for each sample.

### Sample preparation, lysis and DNA extraction

After all leaves were measured and photographed, the five leaves belonging to each individual plant were pooled in 15 ml sterile sample tubes containing 96% denatured ethanol and stored at ~ 5 °C. The ethanol supernatant of all 30 samples was carefully removed immediately before shipment to the Botanische Staatssammlung Munich (SNSB-BSM, Germany) for further processing (export permit WT2020-001235), where 96% denatured ethanol was re-added to the samples. Prey items were separated from the leaves in order to reduce the amount of plant tissue per sample relative to the amount of insect tissue (*Drosera* leaf tissue is rich in polyphenols and polysaccharides which are known to interfere with DNA extraction and amplification^34^); for this, prey items still attached to the leaves were carefully detached from the leaves using forceps under a stereomicroscope, and prey items were transferred into 2 ml lysis cups that were filled with 96% denatured ethanol. Therefore, most of the leaf tissue (except for the tentacles) was removed before lysis.

For better lysis and DNA extraction of the insect tissue, samples were subsequently homogenised for 30–60 s using a FastPrep96 (MP Biomedicals) with addition of sterile steel beads. DNA extraction was conducted following the protocol of Ivanova et al.^35^. Briefly, 200 µl of insect lysis buffer with proteinase K in a 1:20 ratio was added to the sample. Polyvinylpyrrolidone was added (until 2% concentration in the solution) to block inhibiting substances such as polyphenols. Samples were incubated overnight at a temperature of 56 °C and lysates were frozen before extraction.

### DNA amplification and metabarcoding

DNA metabarcoding was conducted at the AIM Lab (AIM—Advanced Identification Methods GmbH, Leipzig, Germany), following the methodology of Morinière et al.^16^, Hardulak et al.^21^, and Hausmann et al.^22,36^.

From each sample, 5 µl of extracted total DNA was used for PCR, along with Plant MyTAQ (Bioline, Luckenwalde, Germany) and High Throughput Sequencing (HTS) adapted mini-barcode primers targeting the mitochondrial cytochrome c oxidase subunit I (CO1-5P) (primers and amplification following Morinière et al.^16^). Amplification success and fragment lengths were verified by gel electrophoresis. Amplified DNA was cleaned up using a 1% sodium acetate and 70% ethanol precipitation method^37^ and resuspended in 50 µl purified water for each sample before proceeding. Illumina Nextera XT (Illumina Inc., San Diego, USA) indices were ligated to the samples in a second PCR reaction applying the same annealing temperature as for the first PCR reaction but with only seven cycles, and ligation success was confirmed by gel electrophoresis. DNA concentrations were measured using a Qubit fluorometer, and adjusted to 40 µl pools containing equimolar concentrations of 100 ng/µl DNA template each. Pools were purified using MagSi-NGSprep Plus (Steinbrenner Laborsysteme GmbH, Wiesenbach, Germany) beads. A final elution volume of 20 µl was used. High Throughput Sequencing (HTS) was performed on an Illumina MiSeq (Illumina Inc., San Diego, USA) using v3 chemistry (2 × 300 basepairs, 600 cycles, maximum of 25 million paired-end reads).

### Barcode sequence analysis, processing and OTU identification

FASTQ files were combined and sequence processing was performed with the VSEARCH v2.4.3 suite^38^ and cutadapt v1.14^39^. Since not all of the sequenced samples yielded reverse reads of high enough quality to enable paired-end merging, only forward reads were utilised. Reads were removed in cases where either the number of mismatches was > 5, where the alignment was shorter than 16 basepairs, or where identity percentage of the alignment was < 90%. Forward primers were removed with cutadapt. Quality filtering was done with the fastq_filter program of VSEARCH (fastq_maxee 2, minimum length of 100 bp). Sequences were dereplicated with derep_fulllength, first at the sample level, and then concatenated into one fasta file, which was then dereplicated. Chimeric sequences were filtered out from the large fasta file using uchime_denovo. Remaining sequences were clustered into Operational Taxonomic Units (OTUs) at 97% identity with cluster_size, and an OTU table was created with usearch_global. To reduce false positives, a cleaning step was employed which excluded read counts in the OTU table of less than 0.01% of the total. OTUs were blasted against a custom database downloaded from BOLD (on 03 February 2021) and NCBI GenBank (February 2020), including taxonomy and BIN (Barcode Index Number) information (contained in the BOLD database), by using Geneious (v.10.2.5; Biomatters, Auckland, New Zealand) and following the methods described in Morinière et al.^16^. The resulting csv file which included the OTU ID, BOLD Process ID, BIN, Hit-%-ID value (percentage of overlap similarity (identical base pairs) of an OTU query sequence with its closest counterpart in the database), length of the top BLAST hit sequence, and phylum, class, order, family, genus, and species information for each detected OTU was exported from Geneious and combined with the OTU table generated by the bioinformatic pipeline (Supplementary Data S1). A consensus taxonomy of the two BLASTs (i.e., only showing the highest taxonomic level of agreement among BOLD and NCBI GenBank for each OTU) was used in subsequent analyses (Supplementary Data S1).

### Sample pooling, data exclusion and plausibility control

**Figure 1:**
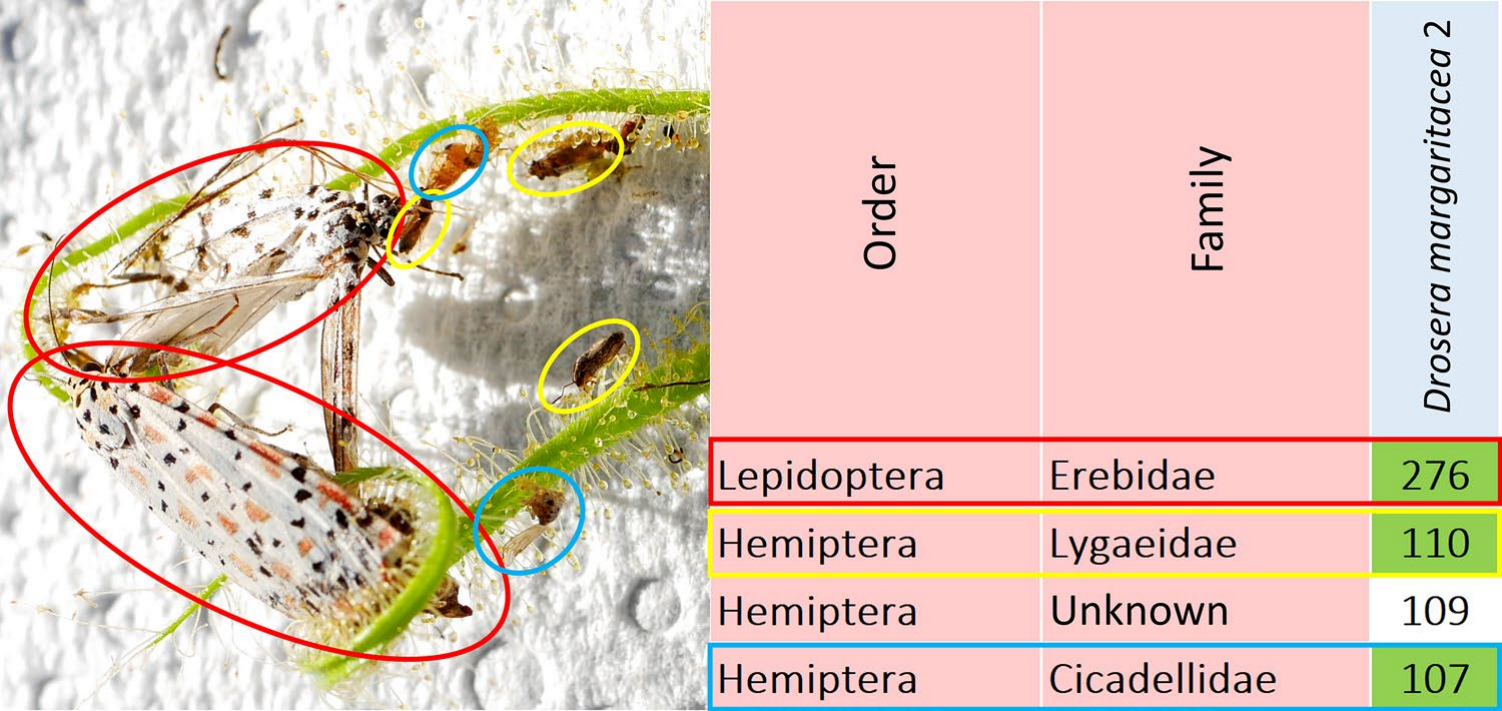
Example of pictorial plausibility control of DNA metabarcoding using *in-situ* macro photography. Left image is a macro photograph of two of the five leaves in sample 2 of *Drosera margaritacea*, on the right is a table of prey families detected by metabarcoding for the same sample showing their read counts in the right-bound column. Only the four prey groups with highest read counts are shown. Colours match detected prey families with visible prey items in the macro photograph (pictorial plausibility control). Picture by T. Krueger.

OTUs were first pooled to the level of arthropod family, as prey spectra analysis was not conducted below this taxonomic level and only 303 of the 739 retrieved OTUs could be identified to genus or below by metabarcoding (Supplementary Data S1). This also resulted in the exclusion of 87 OTUs above the taxonomic level of organismic order. In addition, microorganisms (such as the arthropod intracellular bacteria of the genus *Wolbachia*), marine taxa, fungi and other obvious contaminants (such as *Homo sapiens*–referring to contamination during human handling and processing of samples) were excluded from analysis. The rather ubiquitous phytophagous mealybugs and mites of Pseudococcidae, Trombidiformes and Mesostigmata were not considered to have been captured as prey, but rather because they parasitised the collected plant tissues, and were thus also excluded. The *in-situ* macro photographs obtained during sampling were used as a plausibility control of the prey spectra data generated by metabarcoding. Each taxon in each sample was carefully attempted to be matched with one or several of the prey items visible in the photographs (Fig. 1). This pictorial plausibility control was conducted conservatively, as taxa were only excluded from further analysis if they consisted of large prey animals (such as, for example, wasps, beetles or moths, documented for each case in Supplementary Data S2) which would have been clearly visible in the pictures if they were truly present. Families mostly consisting of small prey animals were generally impossible to confirm or exclude by pictorial plausibility control, as small unidentifiable “crumbs” of prey material were present on most leaves (see^8^). Data on prey spectra composition was compiled and analysed as presence/absence only, because metabarcoding does not allow for accurate estimations of prey quantity^16,26,27^. Finally, the number of samples in which each prey taxon was present was counted for each *Drosera* species, as well as across all three species.

### Statistical analysis

Prey spectra composition was compared between all three species (including all pairwise comparisons) by using analysis of similarity (ANOSIM) in PRIMER 7^40^. After creating Bray–Curtis resemblance matrices, prey spectra dissimilarity was quantified using the ANOSIM R-statistic which ranges from 0 (100% similarity) to 1 (0% similarity)^40^. No data transformations were required, as metabarcoding data were treated as presence/absence only. Subsequently, similarity percentages (SIMPER) were calculated in PRIMER 7 to identify prey groups contributing most to dissimilarity (more than 15% for arthropod orders and the five taxa contributing most to dissimilarity for arthropod families^8^).

Total numbers of captured prey per cm of leaf length (as determined by analysis of *in-situ* prey pictures) were compared between all three species using non-parametric Kruskal–Wallis tests with Dunn-Bonferroni post-hoc pairwise comparisons.

## Results

### Prey spectra detected by DNA metabarcoding

**Figure 2:**
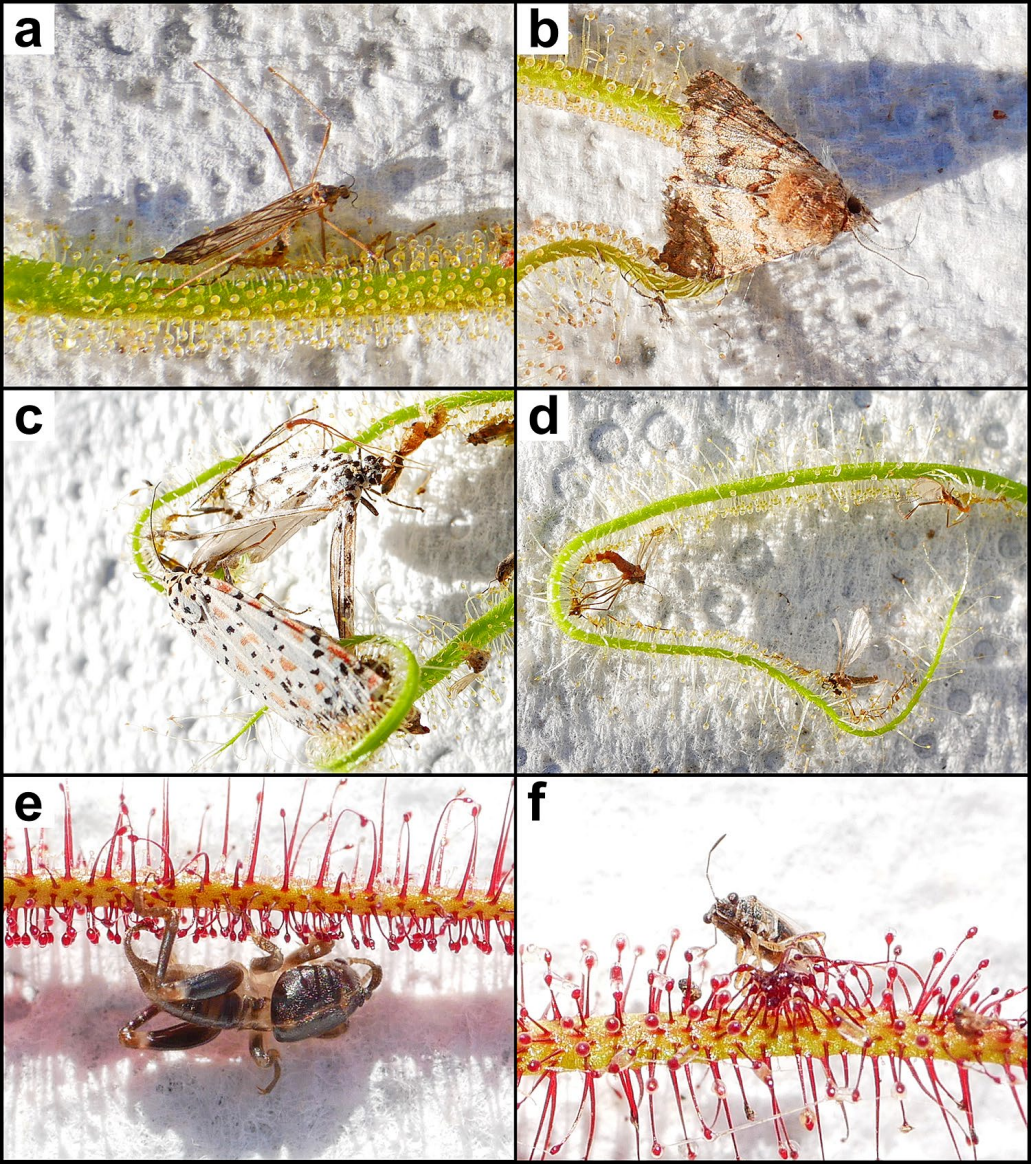
Examples of captured arthropod prey detected and correctly identified by DNA metabarcoding in three Western Australian species of *Drosera* sect. *Arachnopus*. The lowest taxonomic level determined by metabarcoding and the corresponding family, order and BOLD Barcode Index Number (BIN) is indicated. (**a**) *Symplecta* sp. (Limoniidae, Diptera, BOLD:AAF8963) captured by *D. finlaysoniana* (Sample 5). (**b**) *Praxis marmarinopa* (Erebidae, Lepidoptera, BOLD:AAC9474) captured by *D. finlaysoniana* (Sample 9). (**c**) 2 individuals of *Utetheisa lotrix* (Erebidae, Lepidoptera, BOLD:AAA4528) captured by *D. margaritacea* (Sample 2). (**d**) Cecidomyiidae (Diptera, BOLD:ACK2565) captured by *D. margaritacea* (Sample 9). (**e)** Early instar nymph of *Gryllotalpa pluvialis* (Gryllotalpidae, Orthoptera, BOLD:AAF7358) captured by *D. hartmeyerorum* (Sample 1). (**f**) *Nysius plebeius* (Lygaeidae, Hemiptera, BOLD:AAI3382) captured by *D. hartmeyerorum* (Sample 7). All pictures by T. Krueger.

**Figure 3:**
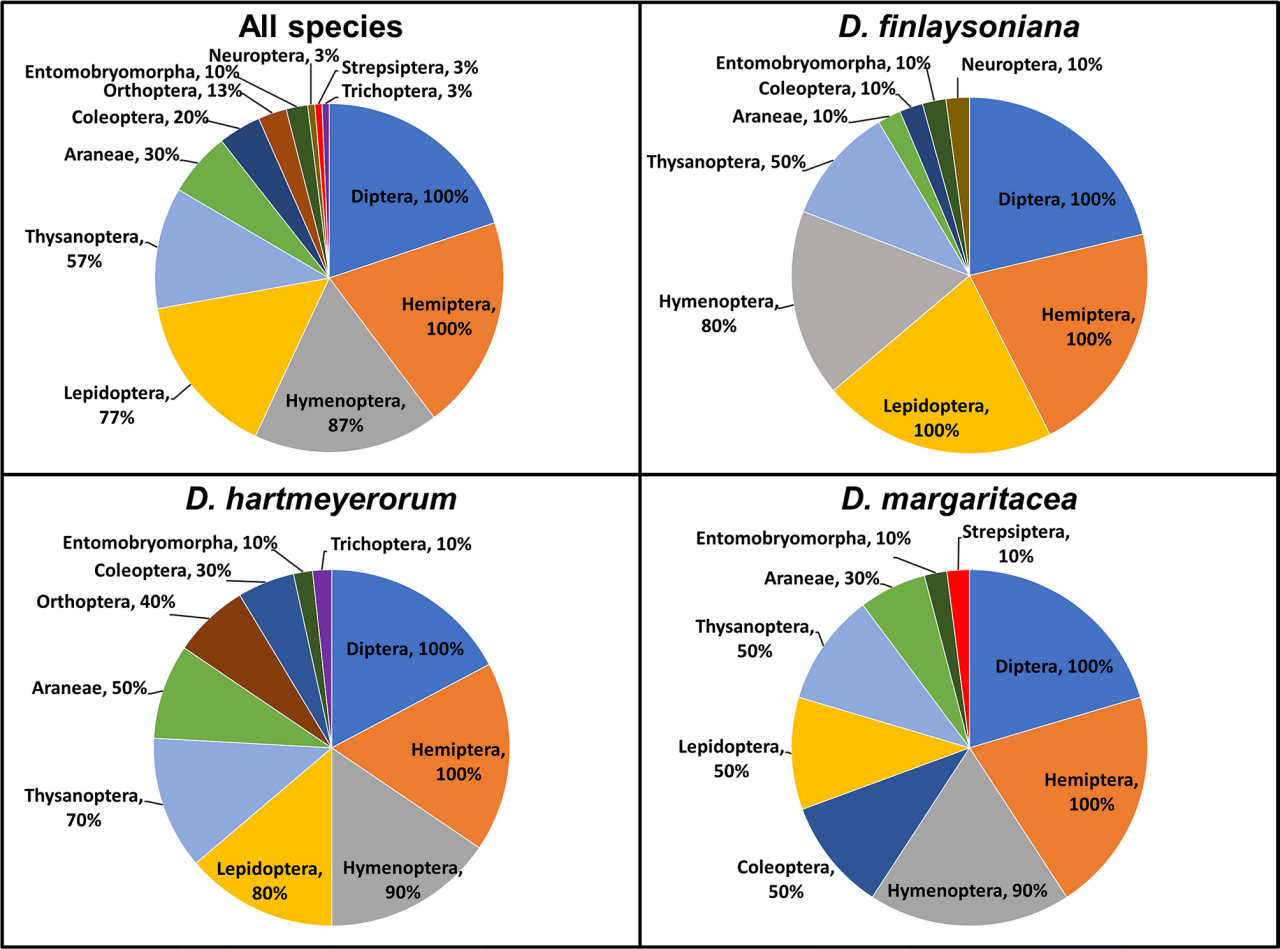
Arthropod orders comprising the prey spectra of three species from *Drosera* sect. *Arachnopus* as detected by DNA metabarcoding. The percentage numbers denote the proportion of *Drosera* samples in which each arthropod order was detected.

DNA metabarcoding confirmed 92 arthropod families belonging to 12 orders caught as prey across all 30 *Drosera* samples (Supplementary Table S1; Figs. 2, 3). Hits from an additional 25 arthropod families were excluded by pictorial plausibility control, most of them detected in *D. hartmeyerorum* samples 1, 4 and 9 (Supplementary Data S2). We found no instance of any prey item being clearly identifiable in the macro photographs but not present in the barcoding data. Of the 739 retrieved OTUs, 71%, 41% and 17% were identified to family-, genus- and species-level, respectively (Supplementary Data S1).

Curculionidae was the family most commonly excluded by pictorial plausibility control as these characteristic weevil beetles were clearly not present, at least as prey (but possibly as eggs in the plant tissue in the case of phytophagous species), in nine of the thirteen samples where they were detected by metabarcoding (in the remaining four samples they were either confirmed by the pictorial plausibility control or not excluded with certainty; see Supplementary Data S2).

Ten of the twelve detected arthropod orders were insects, with only Araneae (spiders, Arachnida, present in 30% of total samples) and Entomobryomorpha (springtails, Collembola, present in 10% of total samples) not belonging to this class (Fig. 3). These two orders were also the only orders exclusively consisting of non-flying prey. Although some of the captured insect families such as Formicidae (ants, Hymenoptera, present in 17% of samples) and larvae of Gryllotalpidae (mole crickets, Orthoptera, larvae only present in sample 1 of *D. hartmeyerorum*; Fig. 2e) include non-flying prey taxa, in the majority of samples only flying adult insects were detected as prey.

The prey orders Diptera and Hemiptera were confirmed to be present in all 30 samples (100%), while Hymenoptera (87%), Lepidoptera (77%) and Thysanoptera (57%) were detected in more than half of samples (Fig. 3). The most commonly (≥ 50%) detected prey groups were “Other Hemiptera” (i.e., hemipterans which could not be assigned by metabarcoding to any family; present in 97% of samples), Hemiptera–Cicadellidae (83%), “Other Diptera” (73%), Diptera–Cecidomyiidae (70%) and Hemiptera–Lygaeidae (70%; Fig. 2f; Supplementary Table S1).

Prey families detected in more than 50% of samples in each of the three species were Diptera–Cecidomyiidae (60–90%; Fig. 2d) and Hemiptera–Cicadellidae (50–90%; Supplementary Table S1). “Other Hemiptera” were present in 90–100% of samples of each species, but the data did not allow for exact identification to family-level.

ANOSIM indicated that differences in the prey spectra between the three species were highly significant at prey family-level (R = 0.784, *P* < 0.001) but non-significant at the level of order (R = 0.079, *P* = 0.063). Additionally, all three species-pairwise comparisons at prey family-level were significant, with the highest R value observed in the comparison between *D. margaritacea* and *D. finlaysoniana* (ANOSIM R = 0.918, *P* < 0.001; Table 2). The only significant pairwise comparison at prey order-level was *D. margaritacea*–*D. finlaysoniana* (ANOSIM R = 0.134, *P* = 0.046; Table 2). SIMPER analysis indicated that no single prey family contributed more than 5% to prey spectra dissimilarity in any of the three pairwise comparisons (Table 2). Aleyrodidae (Hemiptera) contributed most to dissimilarity in both pairwise comparisons involving *D. margaritacea* (this prey family was detected in much more samples of this species), while Lygaeidae had the highest contribution in the SIMPER comparison of *D. finlaysoniana* and *D. hartmeyerorum* (where it was more commonly detected in the latter species; Table 2). However, the individual contributions to dissimilarity of most prey families were generally very similar within the pairwise species comparisons (Table 2). When analysed at order-level, Lepidoptera contributed most to prey dissimilarity in both the *D. margaritacea*–*D. finlaysoniana* and *D. margaritacea*–*D. hartmeyerorum* comparisons (in both cases detected much less commonly in the *D. margaritacea* samples) but did not contribute more than 15% to dissimilarity in the *D. finlaysoniana*–*D. hartmeyerorum* comparison (Table 2). SIMPER analysis further indicated that all pairwise comparisons among species showed higher average dissimilarity than samples of the same species.

**Table 2:** DNA metabarcoding detection of family- and order-level prey spectra differences among three species from *D.* sect. *Arachnopus* in Western Australia.

	Pairwise *Drosera* species comparison	ANOSIM R	*P*	5 Prey families contributing most to dissimilarity in SIMPER analysis (contribution in %; species in which prey family was more commonly detected)
Family-level	*D. margaritacea–D. finlaysoniana*	0.918	< 0.001	Aleyrodidae (4.46; *D. m.*) Chironomidae (4.01; *D. f.*) Other Diptera (3.62; *D. f.*) Muscidae (3.50; *D. f.*) Syrphidae (3.25; *D. f.*)
	*D. margaritacea–D. hartmeyerorum*	0.749	< 0.001	Aleyrodidae (4.15; *D. m.*) Lygaeidae (4.11; *D. m.*) Torymidae (3.59; *D. m.*) Muscidae (3.51; *D. h.*) Other Diptera (3.47; *D. h.*)
	*D. finlaysoniana–D. hartmeyerorum*	0.642	< 0.001	Lygaeidae (3.59; *D. f.*) Chironomidae (3.47; *D. f.*) Syrphidae (3.47; *D. f.*) Calliphoridae (3.12; *D. f.*) Sarcophagidae (3.08; *D. f.*)
Order-level	*D. margaritacea–D. finlaysoniana*	0.134	0.046	Lepidoptera (20.38; *D. f.*) Thysanoptera (20.12; N/A) Coleoptera (19.62; *D. m.*)
	*D. margaritacea–D. hartmeyerorum*	0.033	0.264	Lepidoptera (17.54; *D. h.*) Thysanoptera (17.49; *D. h.*) Coleoptera (17.16; *D. m.*) Araneae (16.57; *D. h.*)
	*D. finlaysoniana–D. hartmeyerorum*	0.046	0.196	Thysanoptera (20.51; *D. h.*) Araneae (18.38; *D. h.*) Orthoptera (15.15; *D. h.*)

Prey compositions are compared by Analysis of Similarity (ANOSIM) and Similarity Percentages (SIMPER) for all pairwise comparisons of studied species.

*D. f.* = *D. finlaysoniana*, *D. h.* = *D. hartmeyerorum*, *D. m.* = *D. margaritacea.*

### Observed total numbers of captured prey

**Figure 4:**
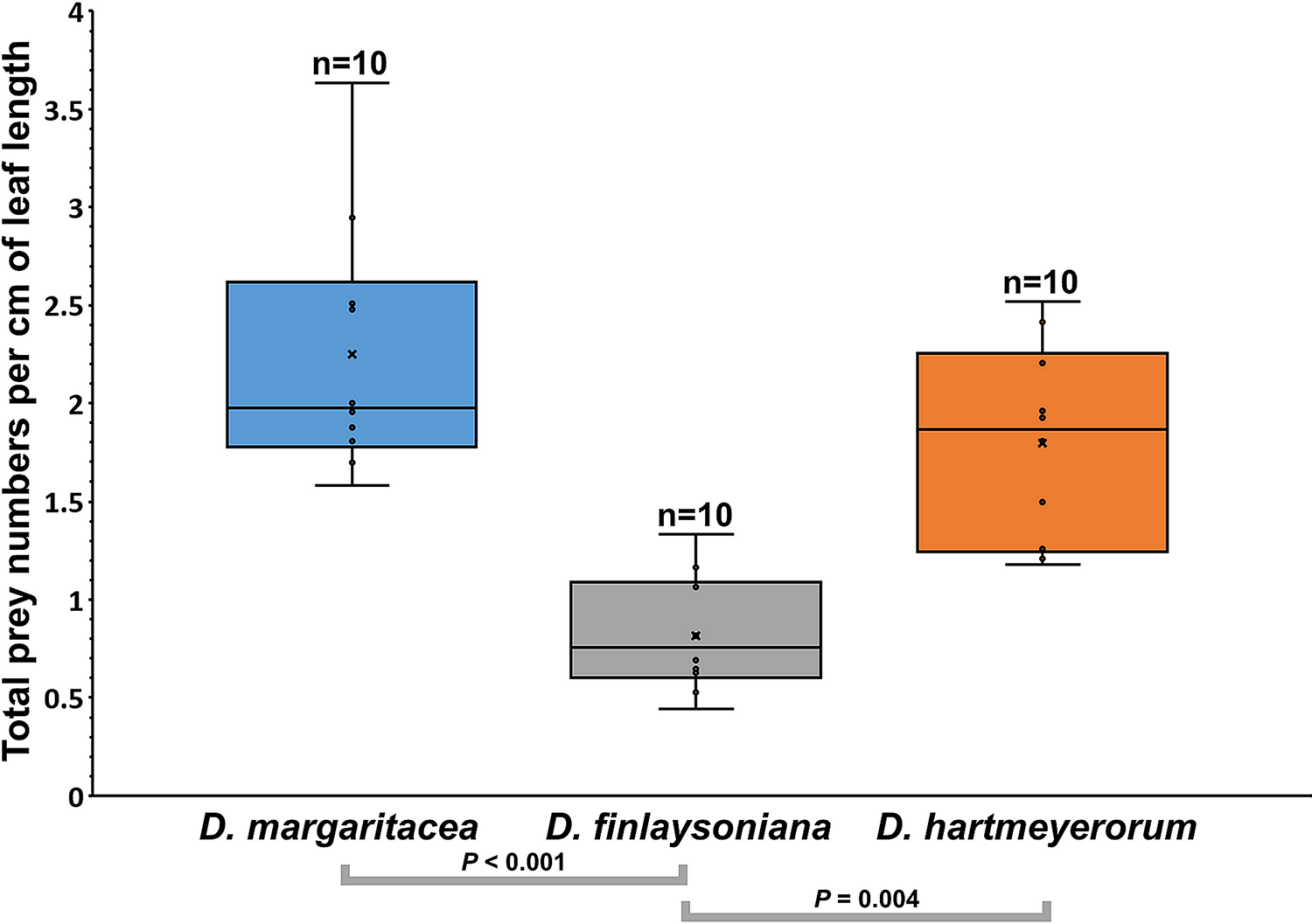
Total prey numbers per cm of leaf length in three species of *Drosera* sect. *Arachnopus* in Western Australia. Grey brackets indicate significant differences between species.

Total prey capture per cm of leaf length, as observed by counting prey items in the *in-situ* macro photographs, did differ significantly among all three studied *Drosera* species (Kruskal–Wallis test, H = 19.19, *P* < 0.001) and in the two pairwise comparisons *D. margaritacea*–*D. finlaysoniana* (*P* < 0.001) and *D. finlaysoniana*–*D. hartmeyerorum* (*P* = 0.004). Prey numbers did not differ in the comparison *D. margaritacea*–*D. hartmeyerorum* (*P* = 0.966). Among the three species, *D. margaritacea* featured the highest average number (2.25 ± 0.65) of prey items per cm of leaf length (Fig. 4). The average measured leaf length of this species was 7.1 ± 1.3 cm (Supplementary Table S2). For *D. hartmeyerorum*, the average number of prey items per cm of leaf length was 1.80 ± 0.50, with an average leaf length in this species of 5.3 ± 1.1 cm (Fig. 4; Supplementary Table S2). Despite *D. finlaysoniana* having by far the largest leaves (average leaf length of 10.4 ± 0.6 cm; Supplementary Table S2), this species had the lowest observed number of prey items per cm of leaf length of the three species (0.81 ± 0.29; Fig. 4).

## Discussion

### A combined DNA metabarcoding/in-situ macro photography approach to reliably analyse carnivorous plant prey spectra

Results indicate that DNA metabarcoding allows for reliable analysis of prey spectra composition in carnivorous plants at a taxonomic resolution and level of completeness unachievable by traditional morphology-based approaches (as performed, for example, by^4–7,9–11^). Even in remote tropical northern Western Australia, where many (if not most) arthropod species have not yet been accessioned into the BOLD or GenBank barcode reference libraries, this method identified over 90% of obtained OTUs from our sample set; most of them at family-level, but 41% to genus-level, and 17% even down to species rank (Supplementary Data S1). Lekesyte et al.^27^ were able to identify 80% of the analysed prey items found on *D. rotundifolia* in England to species-level. However, their sampling was performed in western Europe, whose entomofauna is comparatively well studied taxonomically and has an excellent coverage in the BOLD reference library of DNA barcodes^41^. New insect barcodes are regularly added to the BOLD library through large-scale initiatives such as the international Barcode of Life Project (iBOL; https://ibol.org/) and its Australian node Australian Barcode of Life Network (ABOLN), hence accuracy of future metabarcoding research performed in Australia can be expected to increase to similar levels soon.

*In-situ* macro photography was found to provide a valuable plausibility control tool for the prey taxa identified by metabarcoding. While many of the smaller prey taxa detected by metabarcoding were impossible to identify in the *in-situ* macro photographs due to their tendency to quickly degenerate after digestion into small, shapeless “crumbs”^8^, this control method considerably reduced the amount of prey taxa detected which were not actually present as prey in the *Drosera* samples. This flaw of metabarcoding is most commonly a consequence of procedural errors resulting in cross-contamination within the DNA extraction procedure^27^, usually resulting in low read numbers. However, *in-situ* macro photographs may also fail to detect species if prey captured by the sundew escaped from the trap^33,42^, or was stolen by larger animals. In both cases, a DNA imprint left on the *Drosera* leaves as excretions, detached scales, hairs or, frequently, as autotomised (shedded) body parts^42^ could have been detected by metabarcoding. Additionally, some barcoding-detected taxa may not constitute prey if they were associated with another captured prey taxon (either as part of its diet, or as a parasite). The latter may explain some barcode hits for taxa not immediately apparent from the *in-situ* macro photographs, as they are (endo)parasites of captured prey taxa. This was likely the case in the detected Strepsiptera (stylops) which are frequently contained as larvae and adult females in their hymenopteran and orthopteran hosts^43^. However, insect endoparasites and other non-obvious prey taxa were by default not excluded by the very conservative approach of pictorial plausibility control. Additionally, in the case of endoparasites, these organisms would also contribute to plant nutrition as “bycatch” after being digested together with their host, despite not having been actively attracted to the carnivorous traps. Finally, the control method tested in this study showed that even heavily digested prey items in the samples had sufficient amounts of intact (mitochondrial) DNA present to be detected by metabarcoding, as we found no instance of any prey item being clearly identifiable in the macro photographs but not present in the barcoding data.

### Prey spectra composition of the studied Drosera species

The analysed prey spectra of the three studied species from *D.* sect. *Arachnopus* most commonly contained flying insects (especially of the orders Diptera and Hemiptera, both present in 100% of the samples; Fig. 3), thus confirming earlier *in-situ* macro photography-based studies of closely-related *D.* sect. *Arachnopus* species by Krueger et al.^8^. All members of *D.* sect. *Arachnopus* are characterised by a large, erect growth habit and thread-like aerial leaves which usually do not contact the ground^8,32^, thereby excluding most ground-dwelling arthropods as prey. This result is also similar to other prey spectra studies of erect-leaved *Drosera* from different geographic areas, where flying insects (particularly Diptera) unanimously comprised almost the entire recorded prey^5,11,44^. Furthermore, this study confirmed the result of Krueger et al.^8^ that Hemiptera—and within this order especially the Cicadellidae—are exceptionally common in the prey spectra of *D.* sect. *Arachnopus* compared with all other, previously studied *Drosera*. A possible explanation for this may be the relatively high abundance of Cicadellidae in tropical habitats^45^ compared to subtropical or temperate habitats where the above-mentioned previous *Drosera* prey spectra studies were conducted.

Of the five most commonly detected orders, Lepidoptera generally comprised the largest prey items in terms of body size or wingspan, respectively. This prey order was exceptionally common in *D. finlaysoniana,* being present in 100% of samples and also visually conspicuous in the *in-situ* photographs. Since this *Drosera* species had by far the largest trapping leaves among the three species studied with an average leaf length of 10.4 ± 0.6 cm (Suppl Appendix S7), and exhibits the largest leaves in *D.* section *Arachnopus*^32^, this may represent an example of large prey items being more easily captured by species with larger trapping leaves^33^. Additionally, the sampled population of *D. finlaysoniana* was huge and dense (see Supplementary Figure S1), probably attracting larger prey and enabling capture of larger prey items by “collective” trapping^46^. Alternatively, Fleischmann^30^ suggested that captured Lepidoptera themselves could attract further individuals of the same species by pheromone release, potentially explaining the very high numbers of this insect order observed in *D. finlaysoniana.*

### Differences among observed prey spectra

Comparison of prey spectra between the three studied *Drosera* species revealed significant differences at arthropod family-level but not at the higher level of arthropod orders, indicating that at a coarse taxonomic resolution, the same five arthropod orders (Diptera, Hemiptera, Hymenoptera, Lepidoptera and Thysanoptera) generally comprise most of the prey in *D.* sect. *Arachnopus*, regardless of given *Drosera* species or habitat. However, as strong differences were discovered in the ANOSIM comparison at family-level, it can be concluded that differences might likely increase with finer taxonomic resolution of prey taxa, a conclusion also reached by the carnivorous plant prey spectra meta-analysis of Ellison & Gotelli^47^. While these differences may be partially attributed to different morphological traits of the three species such as leaf scent^8,30^ or eglandular appendages^31^, the very high ANOSIM R-values returned and the large number of prey families contributing nearly equally to dissimilarity (Table 2) indicate that the most likely explanation is very different available prey spectra at the three study sites. Indeed, significant differences among different study sites, even within the same species, were previously reported for *Drosera rotundifolia* by Lekesyte et al.^27^ and for four species from *D.* sect. *Arachnopus* by Krueger et al.^8^. Notably, the three study sites feature different habitat types and climate regimes (Supplementary Fig. S1).

Analyses indicate that there is likely little specialisation in prey capture by the three studied *Drosera* species. For example, the relatively high detection rate of Lepidoptera in the samples of *D. finlaysoniana* and *D. hartmeyerorum* compared to *D. margaritacea* may be explained by the lake margin habitats of the former two species, while the latter species was found in a completely dry drainage channel lacking any nearby waterbodies (Supplementary Fig. S1). Lepidoptera are likely to occur in much higher concentrations near water sources, especially during the dry season (May to November) when the surrounding areas are lacking other water sources (G. Bourke in Fleischmann^30^).

### Estimating prey quantity

In addition to providing a plausibility control for the compositional prey analysis by metabarcoding, the *in-situ* macro photography method facilitated an estimation of prey quantity per sample. Metabarcoding by itself is currently not a reliable tool for prey quantification due to the lack of a linear relationship between the number of sequence reads and organism biomass^26,27^.

In contrast to Krueger et al.^8^, who generally found more prey items on larger trapping leaves in species of *D.* sect. *Arachnopus* (even when values were compared as per cm of trapping leaf length), the species with the largest leaves studied here (*D. finlaysoniana*) captured significantly less prey items than the smaller-leaved species *D. margaritacea* and *D. hartmeyerorum* (Fig. 4). However, while Krueger et al.^8^ was able to compare sympatric species (thus minimising any potential effects of the habitat or region on prey spectra), the three species in this study were studied at three different, geographically distant sites. While it is possible that overall prey abundance in the habitat was much lower at the *D. finlaysoniana* study site (Site 1), it can be hypothesised that the low total prey capture observed in this species may be due to the very large and extremely dense population resulting in strong intraspecific competition for prey (see Supplementary Fig. S1). This effect of population structure on prey capture has also been observed by Gibson^48^ and Tagawa and Watanabe^46^ who found a significant negative correlation between total prey capture and population density in different species of *Drosera*.

### Conclusions and outlook

Our study is the first to employ a DNA metabarcoding approach supported by controls for species presence to analyse carnivorous plant prey spectra. When combined with *in-situ* macro photography, this method is clearly superior in terms of taxonomic resolution and completeness for analysis of environmental bulk samples (containing different organisms in highly variable states of preservation), as used here for the reconstruction of prey spectra of carnivorous plants. The capability of this method increases with new reference barcodes being regularly added to DNA barcode libraries (such as BOLD and NCBI GenBank) and it thus has the potential to become the standard methodology for future carnivorous plant prey spectra research.

Additional studies are needed to test this method for other carnivorous plant species and genera, especially those possessing different trap types. Within Western Australia, three additional trap types occur: snap traps (*Aldrovanda*), suction traps (*Utricularia*) and pitfall traps (*Cephalotus*). In particular, it might be expected that *in-situ* macro photography will not work as well for the extremely small, typically submerged traps of *Aldrovanda* and *Utricularia* (which also completely enclose their captured, microscopic prey items^49^), potentially necessitating usage of alternative control methods for metabarcoding data. Furthermore, even within *Drosera* (adhesive traps) some species may require adjustments to the methodology presented here as they accumulate captured prey in a central point via tentacle movement (e.g., many climbing tuberous *Drosera*) or their leaves may be very difficult to place on paper sheets with the sticky side facing upwards (e.g., all pygmy *Drosera*). The latter problem may be solved by using reverse action forceps and photographing the leaves while held in place by the forceps.

Extensive sampling of sites with co-occurring species from *D.* sect. *Arachnopus* is clearly required to better understand the ecological role of trap scent and eglandular appendages in this section. For example, manipulation experiments involving the removal of all yellow blackberry-shaped appendages of *D. hartmeyerorum* (which have been hypothesised to function as visual prey attractants^31^) and subsequent metabarcoding prey spectra comparisons of mutilated plants lacking emergences with control plants are proposed. Potential effects of population density on prey spectra (as hypothesised here for *D. finlaysoniana*) could be studied by comparing prey spectra of individual plants from within mass populations with more exposed-growing individuals of the same population.

## Supplementary Information

Supplementary Information 1.

Supplementary Information 2.

Supplementary Information 3.

Supplementary Information 4.
