# Supplementary figures and images for: A novel approach for reliable qualitative and quantitative prey spectra identification of carnivorous plants combining DNA metabarcoding and macro photography
Source: Sci Rep. 2022 Mar 21;12:4778. doi: 10.1038/s41598-022-08580-8 (PMC8938489; doi:10.1038/s41598-022-08580-8)

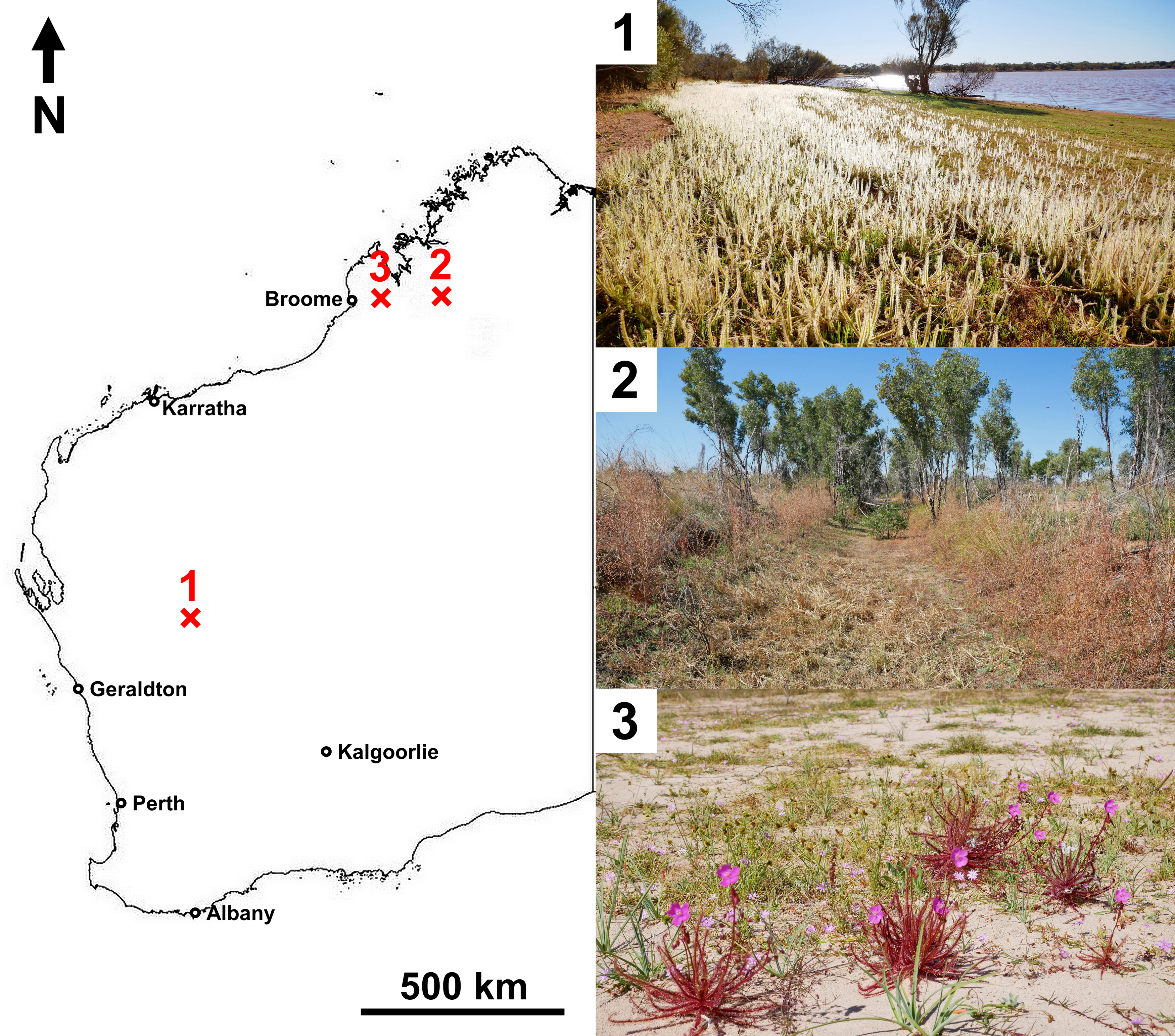

Supplement: Supplementary file 3 — Supplementary Information 3. [file 41598_2022_8580_MOESM3_ESM.tif]
