# Supplementary material for: A novel approach for reliable qualitative and quantitative prey spectra identification of carnivorous plants combining DNA metabarcoding and macro photography
Source: Sci Rep. 2022 Mar 21;12:4778. doi: 10.1038/s41598-022-08580-8 (PMC8938489; doi:10.1038/s41598-022-08580-8)
Supplement: Supplementary file 4 — Supplementary Information 4. [file 41598_2022_8580_MOESM4_ESM.docx]

**Supplementary material**

**A novel approach for reliable qualitative and quantitative prey spectra identification of carnivorous plants combining DNA metabarcoding and macro photography**

Thilo Krueger, Adam T. Cross, Jeremy Hübner, Jérôme Morinière, Axel Hausmann, Andreas Fleischmann


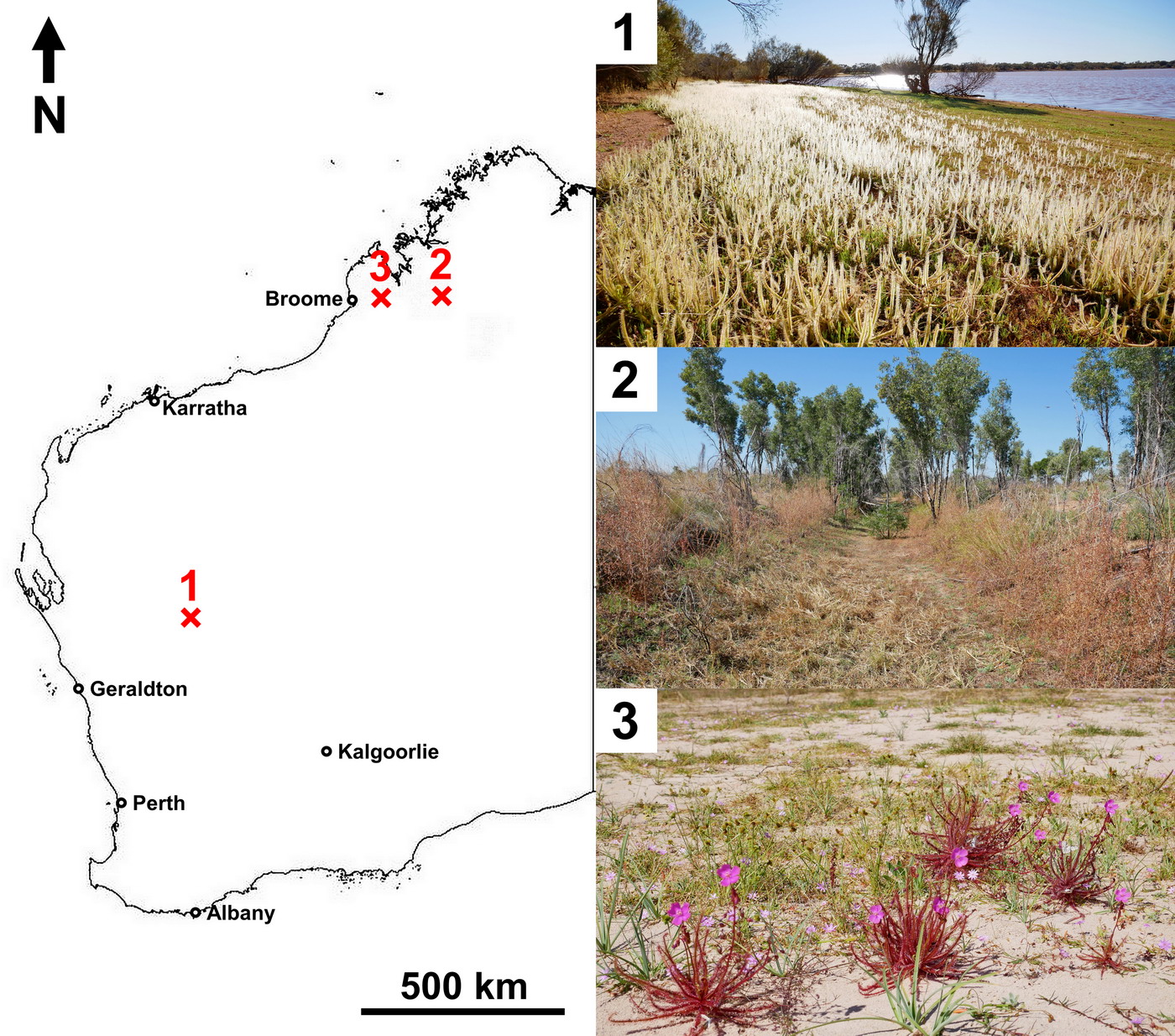


**Supplementary Figure S1.** **Locations and habitats of the three study sites in Western Australia.** For each site a picture of the habitat is provided. Site 1 featured a large and very dense population of *Drosera finlaysoniana* adjacent to a freshwater lake near Cue. Site 2 is located in a narrow artificial drainage channel which was completely dry at the time of study and only ca. 100 plants of *D.* *margaritacea* were found to sparsely populate this habitat. Site 3 featured a large but scattered population of *D. hartmeyerorum* (red plants visible in foreground) growing in sand near an extensive wetland system. Photographs by T. Krueger. Map created in Google Earth Pro version 7.3 (https://www.google.com/earth/versions/) using Australian coastline dataset "GEODATA COAST 100K 2004" available from http://pid.geoscience.gov.au/dataset/ga/61395 (Creative Commons Attribution 4.0 International Licence).

**Supplementary Table S1. Arthropod taxa captured by the three studied species of *Drosera* sect. *Arachnopus* from Western Australia.** Total numbers and percentages of samples where each prey group was detected by DNA metabarcoding are indicated. Arthropod orders are presented in bold.

| Prey group | | All 3 *Drosera* species | Present in % of samples | *D. finlaysoniana* | Present in % of samples | *D. hartmeyerorum* | Present in % of samples | *D. margaritacea* | Present in % of samples |  |
| --- | --- | --- | --- | --- | --- | --- | --- | --- | --- | --- |
| Araneae (Arachnida) | | **9** | **30%** | **1** | **10%** | **5** | **50%** | **3** | **30%** |  |
|  | Linyphiidae | 1 | 3% | 0 | 0% | 1 | 0 | 0 | 0% |  |
|  | Lycosidae | 1 | 3% | 0 | 0% | 1 | 0 | 0 | 0% |  |
|  | Oxyopidae | 1 | 3% | 1 | 10% | 0 | 0 | 0 | 0% |  |
|  | Pisauridae | 1 | 3% | 0 | 0% | 1 | 0 | 0 | 0% |  |
|  | Other Araneae | 5 | 17% | 0 | 0% | 2 | 3 | 3 | 30% |  |
| Coleoptera | | **6** | **20%** | **1** | **10%** | **3** | **30%** | **5** | **50%** |  |
|  | Brentidae | 1 | 3% | 0 | 0% | 0 | 1 | 1 | 10% |  |
|  | Cantharidae | 1 | 3% | 1 | 10% | 0 | 0 | 0 | 0% |  |
|  | Chrysomelidae | 2 | 7% | 0 | 0% | 2 | 0 | 0 | 0% |  |
|  | Coccinellidae | 2 | 7% | 0 | 0% | 1 | 1 | 1 | 10% |  |
|  | Curculionidae | 4 | 13% | 0 | 0% | 0 | 4 | 4 | 40% |  |
|  | Other Coleoptera | 1 | 3% | 0 | 0% | 1 | 0 | 0 | 0% |  |
| Diptera | | **30** | **100%** | **10** | **100%** | **10** | **100%** | **10** | **100%** |  |
|  | Agromyzidae | 2 | 7% | 1 | 10% | 1 | 0 | 0 | 0% |  |
|  | Anthomyiidae | 5 | 17% | 1 | 10% | 4 | 0 | 0 | 0% |  |
|  | Bibionidae | 1 | 3% | 1 | 10% | 0 | 0 | 0 | 0% |  |
|  | Calliphoridae | 6 | 20% | 6 | 60% | 0 | 0 | 0 | 0% |  |
|  | Canacidae | 2 | 7% | 2 | 20% | 0 | 0 | 0 | 0% |  |
|  | Cecidomyiidae | 21 | 70% | 6 | 60% | 6 | 9 | 9 | 90% |  |
|  | Ceratopogonidae | 5 | 17% | 1 | 10% | 4 | 0 | 0 | 0% |  |
|  | Chironomidae | 10 | 33% | 8 | 80% | 2 | 0 | 0 | 0% |  |
|  | Chloropidae | 3 | 10% | 2 | 20% | 1 | 0 | 0 | 0% |  |
|  | Culicidae | 1 | 3% | 0 | 0% | 0 | 1 | 1 | 10% |  |
|  | Dolichopodidae | 2 | 7% | 2 | 20% | 0 | 0 | 0 | 0% |  |
|  | Drosophilidae | 5 | 17% | 5 | 50% | 0 | 0 | 0 | 0% |  |
|  | Ephydridae | 4 | 13% | 0 | 0% | 1 | 3 | 3 | 30% |  |
|  | Fanniidae | 1 | 3% | 0 | 0% | 0 | 1 | 1 | 10% |  |
|  | Heleomyzidae | 1 | 3% | 0 | 0% | 1 | 0 | 0 | 0% |  |
|  | Limoniidae | 6 | 20% | 5 | 50% | 0 | 1 | 1 | 10% |  |
|  | Muscidae | 14 | 47% | 7 | 70% | 7 | 0 | 0 | 0% |  |
|  | Mycetophilidae | 3 | 10% | 1 | 10% | 2 | 0 | 0 | 0% |  |
|  | Phoridae | 5 | 17% | 1 | 10% | 2 | 2 | 2 | 20% |  |
|  | Pipunculidae | 2 | 7% | 0 | 0% | 1 | 1 | 1 | 10% |  |
|  | Psychodidae | 3 | 10% | 2 | 20% | 0 | 1 | 1 | 10% |  |
|  | Sarcophagidae | 10 | 33% | 7 | 70% | 2 | 1 | 1 | 10% |  |
|  | Scathophagidae | 1 | 3% | 1 | 10% | 0 | 0 | 0 | 0% |  |
|  | Sciaridae | 6 | 20% | 2 | 20% | 3 | 1 | 1 | 10% |  |
|  | Sphaeroceridae | 3 | 10% | 2 | 20% | 1 | 0 | 0 | 0% |  |
|  | Stratiomyidae | 1 | 3% | 1 | 10% | 0 | 0 | 0 | 0% |  |
|  | Syrphidae | 8 | 27% | 7 | 70% | 0 | 1 | 1 | 10% |  |
|  | Tachinidae | 4 | 13% | 2 | 20% | 2 | 0 | 0 | 0% |  |
|  | Tephritidae | 2 | 7% | 2 | 20% | 0 | 0 | 0 | 0% |  |
|  | Tipulidae | 6 | 20% | 0 | 0% | 6 | 0 | 0 | 0% |  |
|  | Other Diptera | 22 | 73% | 10 | 100% | 9 | 3 | 3 | 30% |  |
| Entomobryomorpha (Collembola) | | **3** | **10%** | **1** | **10%** | **1** | **10%** | **1** | **10%** |  |
| Hemiptera | | **30** | **100%** | **10** | **100%** | **10** | **100%** | **10** | **100%** |  |
|  | Aleyrodidae | 10 | 33% | 0 | 0% | 1 | 9 | 9 | 90% |  |
|  | Aphididae | 5 | 17% | 0 | 0% | 0 | 5 | 5 | 50% |  |
|  | Cicadellidae | 25 | 83% | 5 | 50% | 10 | 100% | 10 | 100% |  |
|  | Delphacidae | 5 | 17% | 0 | 0% | 1 | 10% | 4 | 40% |  |
|  | Issidae | 1 | 3% | 0 | 0% | 1 | 10% | 0 | 0% |  |
|  | Liviidae | 2 | 7% | 0 | 0% | 2 | 20% | 0 | 0% |  |
|  | Lygaeidae | 21 | 70% | 9 | 90% | 2 | 20% | 10 | 100% |  |
|  | Miridae | 6 | 20% | 3 | 30% | 3 | 30% | 0 | 0% |  |
|  | Monophlebidae | 1 | 3% | 0 | 0% | 0 | 0% | 1 | 10% |  |
|  | Psyllidae | 2 | 7% | 0 | 0% | 2 | 20% | 0 | 0% |  |
|  | Triozidae | 1 | 3% | 0 | 0% | 1 | 10% | 0 | 0% |  |
|  | Other Hemiptera | 29 | 97% | 10 | 100% | 10 | 100% | 9 | 90% |  |
| Hymenoptera | | **26** | **87%** | **8** | **80%** | **9** | **90%** | **9** | **90%** |  |
|  | Bethylidae | 1 | 3% | 1 | 10% | 0 | 0% | 0 | 0% |  |
|  | Braconidae | 1 | 3% | 0 | 0% | 1 | 10% | 0 | 0% |  |
|  | Dryinidae | 2 | 7% | 1 | 10% | 1 | 10% | 0 | 0% |  |
|  | Eucharitidae | 2 | 7% | 0 | 0% | 0 | 0% | 2 | 20% |  |
|  | Eulophidae | 1 | 3% | 0 | 0% | 0 | 0% | 1 | 10% |  |
|  | Formicidae | 5 | 17% | 1 | 10% | 2 | 20% | 2 | 20% |  |
|  | Ichneumonidae | 12 | 40% | 5 | 50% | 7 | 70% | 0 | 0% |  |
|  | Mymaridae | 5 | 17% | 1 | 10% | 1 | 10% | 3 | 30% |  |
|  | Platygastridae | 6 | 20% | 0 | 0% | 1 | 10% | 5 | 50% |  |
|  | Pompilidae | 2 | 7% | 0 | 0% | 1 | 10% | 1 | 10% |  |
|  | Torymidae | 9 | 30% | 2 | 20% | 0 | 0% | 7 | 70% |  |
|  | Trichogrammatidae | 4 | 13% | 0 | 0% | 3 | 30% | 1 | 10% |  |
|  | Other Hymenoptera | 14 | 47% | 3 | 30% | 3 | 30% | 8 | 80% |  |
| Lepidoptera | | **23** | **77%** | **10** | **100%** | **8** | **80%** | **5** | **50%** |  |
|  | Cosmopterigidae | 1 | 3% | 0 | 0% | 1 | 10% | 0 | 0% |  |
|  | Crambidae | 10 | 33% | 3 | 30% | 5 | 50% | 2 | 20% |  |
|  | Erebidae | 4 | 13% | 2 | 20% | 1 | 10% | 1 | 10% |  |
|  | Gelechiidae | 4 | 13% | 2 | 20% | 1 | 10% | 1 | 10% |  |
|  | Geometridae | 3 | 10% | 1 | 10% | 2 | 20% | 0 | 0% |  |
|  | Gracillariidae | 1 | 3% | 0 | 0% | 0 | 0% | 1 | 10% |  |
|  | Lycaenidae | 4 | 13% | 4 | 40% | 0 | 0% | 0 | 0% |  |
|  | Noctuidae | 3 | 10% | 2 | 20% | 1 | 10% | 0 | 0% |  |
|  | Oecophoridae | 1 | 3% | 0 | 0% | 0 | 0% | 1 | 10% |  |
|  | Pterophoridae | 7 | 23% | 0 | 0% | 5 | 50% | 2 | 20% |  |
|  | Pyralidae | 1 | 3% | 1 | 10% | 0 | 0% | 0 | 0% |  |
|  | Scythrididae | 3 | 10% | 1 | 10% | 0 | 0% | 2 | 20% |  |
|  | Tineidae | 1 | 3% | 0 | 0% | 1 | 10% | 0 | 0% |  |
|  | Tortricidae | 2 | 7% | 2 | 20% | 0 | 0% | 0 | 0% |  |
|  | Other Lepidoptera | 4 | 13% | 1 | 10% | 3 | 30% | 0 | 0% |  |
| Neuroptera | | **1** | **3%** | **1** | **10%** | **0** | **0%** | **0** | **0%** |  |
|  | Coniopterygidae | 1 | 3% | 1 | 10% | 0 | 0% | 0 | 0% |  |
| Orthoptera | | **4** | **13%** | **0** | **0%** | **4** | **40%** | **0** | **0%** |  |
|  | Acrididae | 2 | 7% | 0 | 0% | 2 | 20% | 0 | 0% |  |
|  | Gryllidae | 1 | 3% | 0 | 0% | 1 | 10% | 0 | 0% |  |
|  | Gryllotalpidae | 1 | 3% | 0 | 0% | 1 | 10% | 0 | 0% |  |
|  | Other Orthoptera | 2 | 7% | 0 | 0% | 2 | 20% | 0 | 0% |  |
| Strepsiptera | | **1** | **3%** | **0** | **0%** | **0** | **0%** | **1** | **10%** |  |
|  | Corioxenidae | 1 | 3% | 0 | 0% | 0 | 0% | 1 | 10% |  |
| Thysanoptera | | **17** | **57%** | **5** | **50%** | **7** | **70%** | **5** | **50%** |  |
|  | Phlaeothripidae | 10 | 33% | 5 | 50% | 3 | 30% | 2 | 20% |  |
|  | Thripidae | 10 | 33% | 0 | 0% | 5 | 50% | 5 | 50% |  |
| Trichoptera | | **1** | **3%** | **0** | **0%** | **1** | **10%** | **0** | **0%** |  |
| Sample size | | **n=30** |  | **n=10** |  | **n=10** |  | **n=10** |  |  |

**Supplementary Table S2. Measured average leaf lengths and observed total prey per cm of leaf length in three sampled species of *D.* sect. *Arachnopus*.**

| **Sample ID/species** | **Average leaf size (cm)** | **Number of captured prey per cm of leaf length** |
| --- | --- | --- |
| margaritacea 1 | 9.0 | 1.96 |
| margaritacea 2 | 7.5 | 2.51 |
| margaritacea 3 | 6.1 | 2.00 |
| margaritacea 4 | 6.3 | 2.48 |
| margaritacea 5 | 8.7 | 3.63 |
| margaritacea 6 | 7.7 | 1.58 |
| margaritacea 7 | 5.5 | 2.95 |
| margaritacea 8 | 8.1 | 1.88 |
| margaritacea 9 | 5.9 | 1.69 |
| margaritacea 10 | 6.2 | 1.81 |
| finlaysoniana 1 | 11.0 | 0.53 |
| finlaysoniana 2 | 10.2 | 0.63 |
| finlaysoniana 3 | 10.7 | 0.82 |
| finlaysoniana 4 | 10.8 | 0.81 |
| finlaysoniana 5 | 9.6 | 1.17 |
| finlaysoniana 6 | 9.3 | 1.33 |
| finlaysoniana 7 | 11.1 | 0.65 |
| finlaysoniana 8 | 10.1 | 0.69 |
| finlaysoniana 9 | 10.7 | 1.07 |
| finlaysoniana 10 | 10.9 | 0.44 |
| hartmeyerorum 1 | 4.9 | 2.20 |
| hartmeyerorum 2 | 7.0 | 1.26 |
| hartmeyerorum 3 | 6.8 | 1.50 |
| hartmeyerorum 4 | 5.4 | 2.52 |
| hartmeyerorum 5 | 3.4 | 2.41 |
| hartmeyerorum 6 | 5.4 | 1.93 |
| hartmeyerorum 7 | 5.2 | 1.81 |
| hartmeyerorum 8 | 5.6 | 1.18 |
| hartmeyerorum 9 | 4.3 | 1.21 |
| hartmeyerorum 10 | 5.4 | 1.96 |
